# Curation of historical phenotypic wheat data from the Czech Genebank for research and breeding

**DOI:** 10.1038/s41597-024-03598-1

**Published:** 2024-07-11

**Authors:** Pavel Svoboda, Vojtěch Holubec, Jochen C. Reif, Marcel O. Berkner

**Affiliations:** 1 Department of Molecular Genetics, Crop Research Institute (CRI), Drnovská 507, 161 06 Prague-6, Ruzyně, Czech Republic; 2 Department of Genebank, Crop Research Institute (CRI), Drnovská 507, 161 06 Prague-6, Ruzyně, Czech Republic; 3 Department of Breeding Research, Leibniz Institute of Plant Genetics and Crop Plant Research (IPK), Gatersleben, Germany

**Keywords:** Data processing, Plant breeding

## Abstract

Climate change and population growth are putting increasing pressure on global food security. The development of high-yielding varieties for important crops such as wheat is crucial to meet these challenges. The basis for this is extensive exploitation of beneficial genetic variation resting in genebanks around the world. Selecting suitable donor genotypes from the vast number of wheat accessions stored in genebanks is a difficult task and depends critically on the density of information on the performance of individual accessions. Therefore, this study aimed to access phenotypic data from the Czech genebank, storing over 13,000 wheat accessions. We curated and analyzed data on heading date, plant height, and thousand grain weight for more than one-third of all available accessions regenerated across 70 years. The data underwent analysis using a linear mixed model, revealing high quality of curated data with heritability reaching 99%. The raw data, but also derived data such as the best linear unbiased estimations, are now available for the wheat collection of the Czech genebank for research and breeding.

## Background & Summary

With a worldwide cultivation area of nearly 220 million ha and a production of about 220 million tons, wheat is one of the most widely grown crops and provides about one-fifth of the calorie and protein intake of the world’s population^1,2^. Wheat supplies about 40% of the dietary intake of essential micronutrients such as zinc, iron, manganese, magnesium, and vitamins B and E for millions of people, who rely on a wheat-based diet^3^, is an important source of energy for livestock^4^, and is processed for various other purposes including fuel^5,6^. Considering the growth rate of human population of 1.2% per year, more efficient wheat varieties will be needed in the future to ensure food security.

An important component in breeding superior varieties is to utilize the variability hidden within collections of genetic resources stored in genebanks around the globe. However, selecting suitable accessions that have the potential to improve desired traits represents a tremendous challenge in this regard, especially considering that the pool of accessions in global genebanks includes hundreds of thousands of individuals and little information is available for individual accessions.

In the Czech Republic, the Genetic Resources Department was launched in 1951, just after the establishment of the Crop Research Institute (CRI). Germplasm collections of the main crops have been gathered and are maintained. Cereals and mainly wheat represent the prevailing share of stored germplasm. The groundbreaking event was the opening of the genebank in 1988, when regeneration cycles prolonged from 5 up to 30 years. The National Program for the Conservation and Use of Genetic Resources of Plants and Agrobiodiversity (NP) was launched in 1993 and led to standardization of processes related to storing and maintaining of genetic resources. Nowadays, the collections include 1,392 species, both cultivated species as well as crop-wild relatives, and 463 genera are represented in total. The total number of accessions maintained in the NP amounts to 56,789, of which wheat accessions accounts for about one fifth. The coordinator of NP is the Genebank Department, CRI Prague-Ruzyně.

The CRI genebank contains 12,598 publicly available accessions of wheat (*Triticum* spp.) recorded in the GRIN Czech documentation system (GRIN Czech, accessed 31 May 2024, https://grinczech.vurv.cz/gringlobal/query/query.aspx). During the period 1951–1988, all accessions were regenerated every 5 to 7 years. This interval was extended to 20–30 years after the introduction of climatized storage in the genebank. Data collected during the regeneration cycles are available only in the form of field notes, but most accessions were subjected to systematic experiments with the aim of characterization and evaluation according to the descriptor list for wheat^7^. The results from these usually three-year trials were averaged, converted to descriptor scale points, and uploaded to the information system EVIGEZ for the period 1980–2015 or GRIN Czech for the period 2015 and later. The raw data of these trials were archived as field notes and published in the form of summaries in the annual or final project reports since 1951. Since 1998, the raw evaluation data have been available in digital form. It is evident that a large amount of evaluation data has been generated from the trials since 1951, but it was available only in the form of mean values in the documentation system. Raw data from the trials were only accessible in person by reviewing the handwritten field books and in the CRI library. These data represent a source of information potentially valuable for wheat breeding, but have not been curated and were not available according to the F(indable) A(ccessible) I(nteroperable) R(eusable) principles for data publications^8^.

Our study relies on historical phenotypic data of spring and winter wheat accessions regenerated in Prague-Ruzyne since the 1950s. The main goal of this study was to curate and publish the historical phenotypic data of the CRI spring and winter wheat collections following the FAIR principles. We focused on three important agronomic traits: heading date (HD), plant height (PH), and thousand grain weight (TGW), which have been generated during the past 70 years.

## Methods

### Plant material

The wheat collection of the Czech genebank, harbored at the Crop Research Institute in Prague, comprises nearly 13,000 accessions within the genus *Triticum*, of which the majority correspond to *Triticum aestivum (GRIN Czech 2024*, entered with following parameters: Accessions Available From a Site; site_acronym: CZE122; Limit: 20,000, accession IDs: 01C01* and 01C02* for winter and spring wheat, respectively). Other cultivated species include the diploid *T. monococcum*, the tetraploids *T. durum*, *T. dicoccum*, *T. polonicum*, *T. isphanicum*, *T. timopheevii*, and *T. carthlicum*, and the hexaploids *T. spelta*, *T. vavilovii*, *T. compactum*, and *T. petropavlovskyi*. In addition, the collection includes wild species at the diploid and tetraploid levels: *T. boeoticum*, *T. urartu*, *T. dicoccoides*, and *T. araraticum*. The phenotypic data presented in this study included 4,534 accessions (1,065 spring wheat and 3,469 winter wheat) which corresponds to more than 1/3 of the entire collection.

### Phenotyping protocol

The main task of genebanks is to conserve genetic material for future generations and provide it for current users, which entails regular regeneration and multiplication in field plots. Seed multiplication is required when (i) seed stocks are no longer sufficient, (ii) germination rates decrease below a critical threshold, (iii) extensive amounts of seeds are demanded by research, or (iv) new accessions are added to the genebank. During the regeneration process, morphological and agronomical traits are scored for the phenotypic comparison with previous regenerations cycles following strict quality guidelines. In case of doubts whether there were any morphological shifts or drifts during propagation, the voucher spike collection kept in the genebank can be used for comparison. Individual accessions were regenerated between 1951 and 2020 in Prague, Ruzyně (latitude 50° 5′ 10.3698″N, longitude 14° 16′ 49.926″E, 364 m.a.s.l., local soil type Orthic Luvisol, 8.5 °C average annual temperature, 510.5 mm average annual rainfall). Not all accessions were regenerated every year, resulting in a non-orthogonal structure of the data.

For seed propagations, spring wheat accessions were usually sown from February to April, while winter wheat accessions were sown from September to October. The accessions were grown in plots with a size 2 m^2^ for regeneration. Evaluation trials consisted of experimental plots with a size of 4 or 10 m^2^ in four replications in completely randomized design. Data were recorded according to the descriptor list for wheat^7^ for the 25–30 traits. The experiments were repeated for 3 (in a few exceptions 2) years in different experimental fields of the genebank. Three traits were selected for this study: heading date (HD), plant height (PH), and thousand grain weight (TGW). HD was assessed for both spring and winter wheat accessions as the number of days from January 1^st^ when 50% of the plants reached heading (BBCH 59)^9^. PH was assessed in cm from the soil surface to the top of spike including awns. TGW was determined after seed harvest and expressed in g on a ~15% grain moisture basis.

### Data analyses

Phenotypic data of each growth type was analyzed separately based on the following mixed model:

1 $${{\boldsymbol{y}}}_{{\boldsymbol{ij}}}={\boldsymbol{\mu }}+{{\boldsymbol{g}}}_{{\boldsymbol{i}}}+{{\boldsymbol{a}}}_{{\boldsymbol{j}}}+{{\boldsymbol{e}}}_{{\boldsymbol{ij}}}$$

where ***y***_***ij***_ stands for observed phenotypic value of the i*th* accession in j*th* year, ***μ*** is the population mean, ***e***_***ij***_ error terms (random), ***g***_***i***_ effect of accessions (fixed), and ***a***_***j***_ effect of the year (random).

ASreml-R^10^ v. 4.1.0.154. was used for the purpose of the analysis and variances of errors were modelled as specific for each year. In a first step, Eq. (1) was used for outlier detection. In order to do that, studentized residuals were used and Bonferroni-Holm tests were applied to correct for multiple testing^11,12^. The outliers were then removed from the dataset and best linear unbiased estimations (BLUEs) for each accession were generated by fitting Eq. (1) on the enhanced data set.

In the next step, the heritability of the main traits was calculated considering both years and accessions as random effects in Eq. (1):

2 $${{\boldsymbol{h}}}^{{\bf{2}}}=\frac{{{\boldsymbol{\sigma }}}_{{\boldsymbol{G}}}^{{\bf{2}}}}{{{\boldsymbol{\sigma }}}_{{\boldsymbol{G}}}^{{\bf{2}}}+\frac{{{\boldsymbol{\sigma }}}_{{\boldsymbol{e}}}^{{\bf{2}}}}{{\boldsymbol{Year}}}}$$

where, ${{\boldsymbol{\sigma }}}_{{\boldsymbol{G}}}^{{\boldsymbol{2}}}$ is genetic variance of the accessions, ${{\boldsymbol{\sigma }}}_{{\boldsymbol{e}}}^{{\boldsymbol{2}}}$ stands for average error variance across regeneration years, ***Year*** is average number of years each accession was tested.

## Data Records

The data analyzed in this study can be accessed in Plant Genomics and Phenomics Research Data Repository (PGP)^13^ and can be found here^14^. Dataset follows the standards of ISA-Tab format to sustain uniform and easy to read description. Original as well as processed/corrected data are supplied.

Information about investigated accessions are provided in two main files (s_winter_wheat.txt and s_spring_wheat.txt). Encompassed information are as follows: (i) National accession identifier, (ii) harvest year, (iii) values for three main traits, (iv) type of values stating whether respective value for main traits is single (sole year of cultivation and one value) or average (one average value based on multiple years of cultivation), (v) geographic origin identifying the country reported by donors or where the respective accessions were collected.

The assay files of the presented study comprise the original historical phenotypic data (“a_spring_wheat.txt” and “a_winter_wheat.txt”) as provided by the CRI genebank that were tested for presence of outliers. By discarding the identified outliers, enhanced data sets were generated (“Outlier.corrected.spring.txt” and “Outlier.corrected.winter.txt”).

**Fig. 1:**
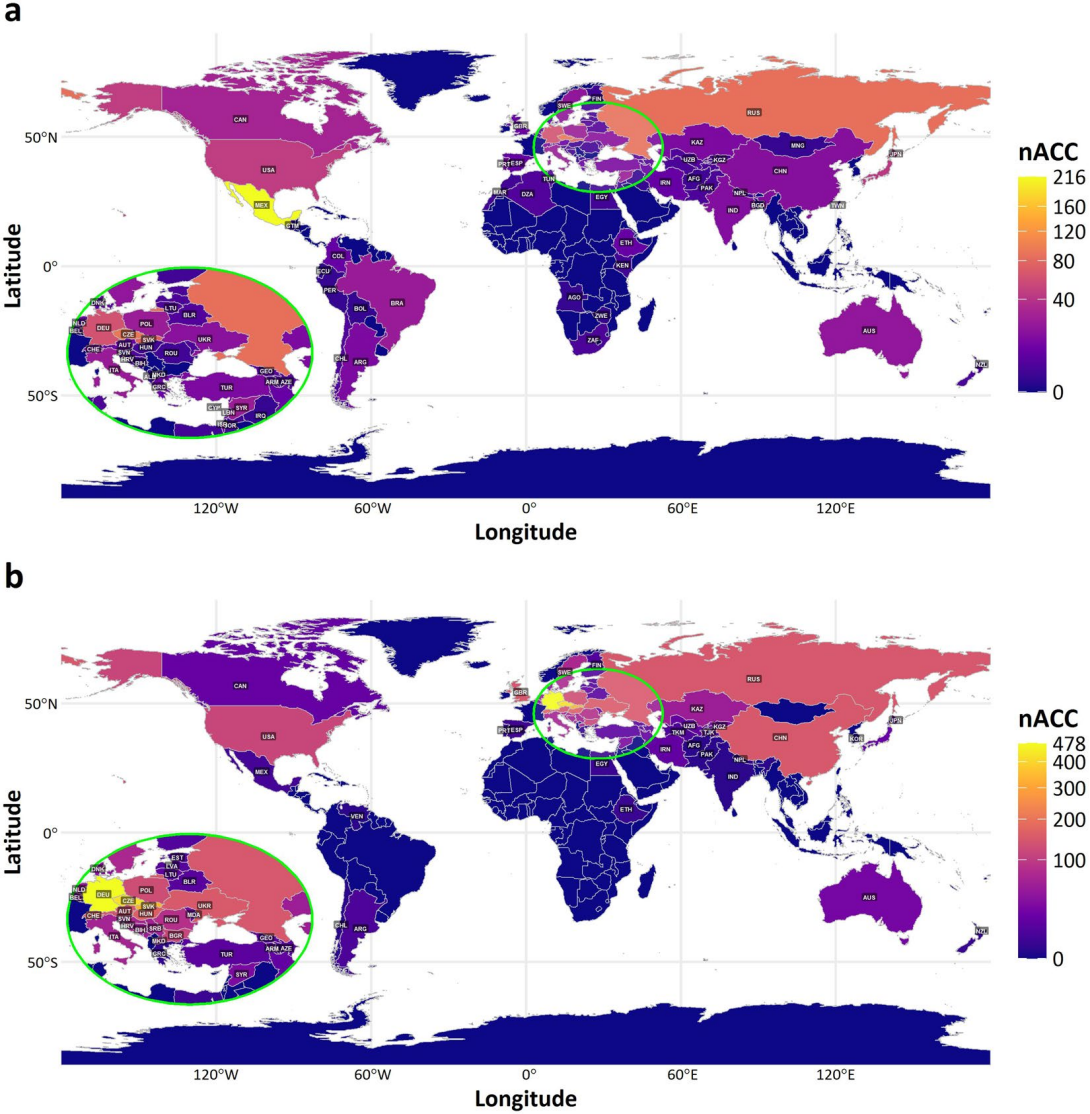
Geographical provenance of (**a**) 1,065 spring and (**b**) 3,469 winter wheat accessions of the collection maintained at the Crop Research Institute (CRI), Prague, Czech Republic, represented within the set of historical phenotypic data. Color of individual countries reflects the number of accessions (nACC) of that particular origin according to color key placed on the right side of each plot. The map inset in the lower left corner magnifies the countries within the highlighted circular area on the map. The three-letter codes display the country name according to the ISO3166 standard.

**Fig. 2:**
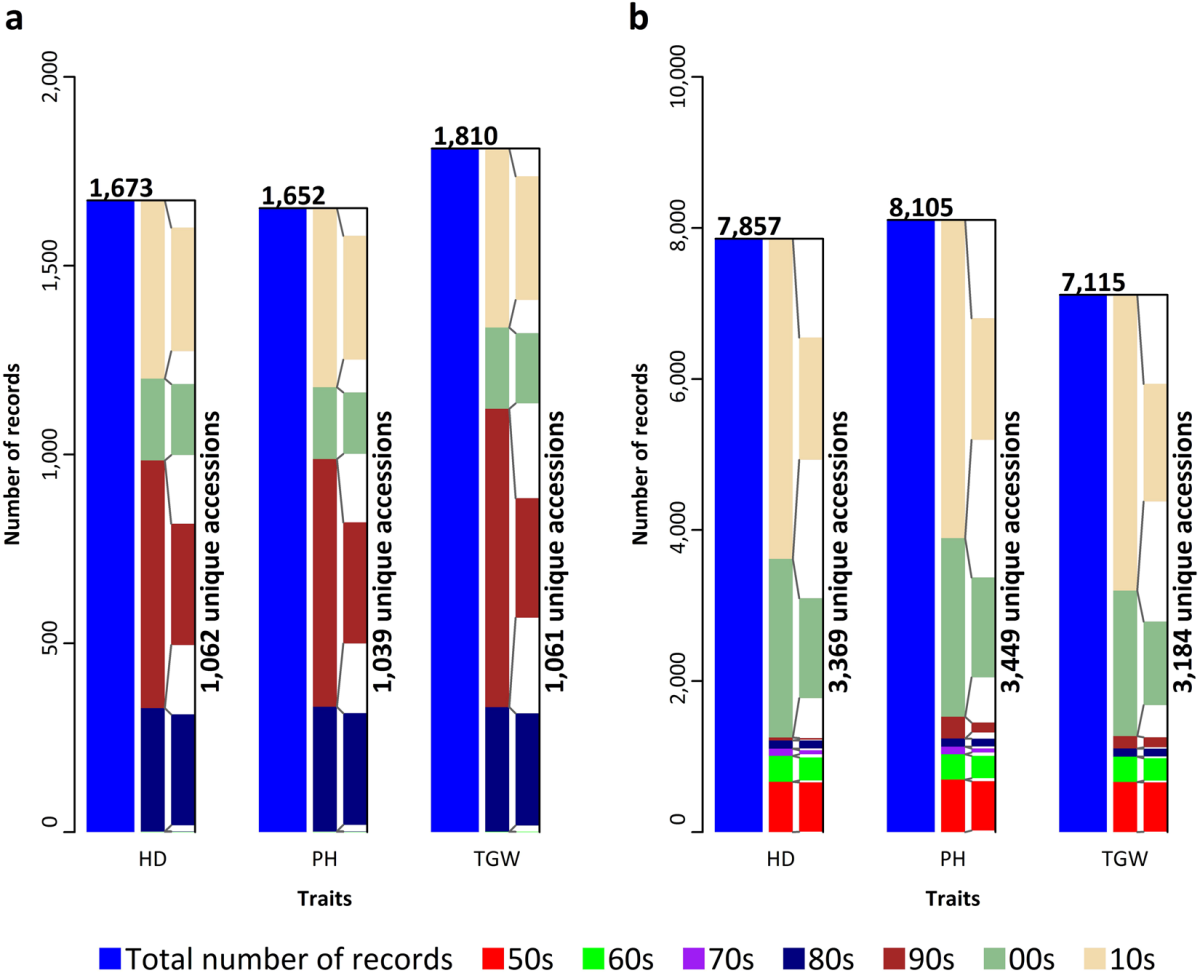
Number of phenotypic records available for heading date (HD), plant height (PH), and thousand grain weight (TGW) for the (**a**) spring and (**b**) winter wheat collections maintained at the Crop Research Institute (CRI), Prague, Czech Republic. Total number of records along with data abundance from individual decades is displayed along with number of accessions covering the phenotypic records.

There are 1,065 unique spring wheat accessions with data for at least one trait in the original records. Mexican accessions are the most represented (20.28%), followed by Russian (7.89%), former Czechoslovakian (6.29%; CSK), German (5.63%), and USA (4.32%) (Fig. 1; Table 1). Additionally, 2.54% of the accessions are of Czech provenance (CZE), which used to be a part of CSK. Therefore, 8.83% of the accessions are of Czech (CZE) or Slovak (SVK) origin. The most data are present for TGW (1,810), followed by HD (1,673) and PH (1,652). The data span 4 consecutive decades, with data from the 1990s and 2010s being the most common (Fig. 2).

**Table 1:** Absolute quantities of 1,065 spring and 3,469 winter wheat accessions maintained at the Crop Research Institute (CRI), Prague, Czech Republic, according to its geographical provenance.

Spring			Winter		
Origin	Accessions (n)	Percentage	Origin	Accessions (n)	Percentage
MEX	216	20.28	DEU	454	13.09
RUS	84	7.89	FRA	297	8.56
CSK	67	6.29	CSK	274	7.9
DEU	60	5.63	CHE	167	4.81
USA	46	4.32	HUN	164	4.73
JPN	43	4.04	CHN	149	4.3
FRA	33	3.1	CZE	148	4.27
CAN	29	2.72	GBR	146	4.21
CZE	27	2.54	UKR	146	4.21
POL	24	2.25	AUT	142	4.09
SYR	24	2.25	RUS	142	4.09
BRA	23	2.16	POL	128	3.69
ITA	23	2.16	BGR	118	3.4
AUS	20	1.88	USA	117	3.37
SWE	20	1.88	ROU	83	2.39
CHN	16	1.5	SWE	73	2.1
IND	15	1.41	ITA	65	1.87
UKR	14	1.31	SVK	62	1.79
ARG	13	1.22	YUG	54	1.56
CHE	13	1.22	NLD	52	1.5
TUR	13	1.22	KAZ	49	1.41
PRT	12	1.13	DNK	41	1.18
ISR	11	1.03	BEL	36	1.04
Others (54)	183	17.17	Others (42)	336	9.73
Unknown	36	3.38	Unknown	26	0.75
Total	1,065	100	Total	3,469	100

Countries represented by less than one percent are comprised under “Others” including 54 and 42 countries of origin for spring wheat and winter wheat, respectively.

Historical data for winter wheat include 3,469 unique accessions, with German accessions being the most abundant (13.09%), followed by French (8.56%), CSK (7.9%), Swiss (4.81%), and Hungarian (4.73%) (Fig. 1; Table 1). More than 4% of the accessions are of Czech origin (CZE). Together with the accessions originating from CSK, there are 12.17% accessions of either CZE or SVK origin. The most abundant data are for PH (8,105), followed by HD (7,857), and TGW (7,115). The data span 7 consecutive decades, with most data from the 2000s and 2010s (Fig. 2).

**Fig. 3:**
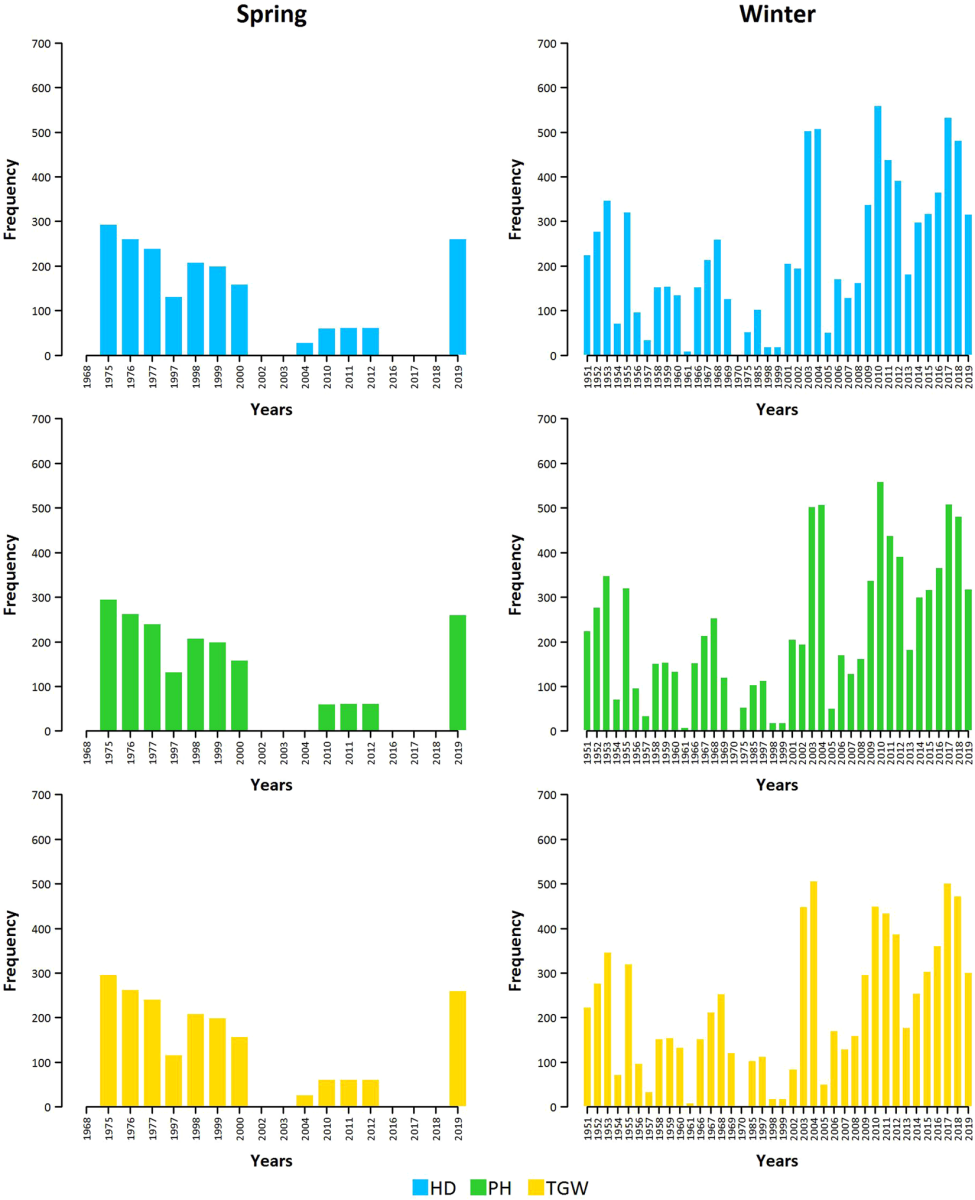
Number of accessions of the spring and winter wheat collections maintained at the Crop Research Institute (CRI), Prague, Czech Republic, that were tested in individual years for heading date (HD), plant height (PH), and thousand grain weight (TGW).

**Fig. 4:**
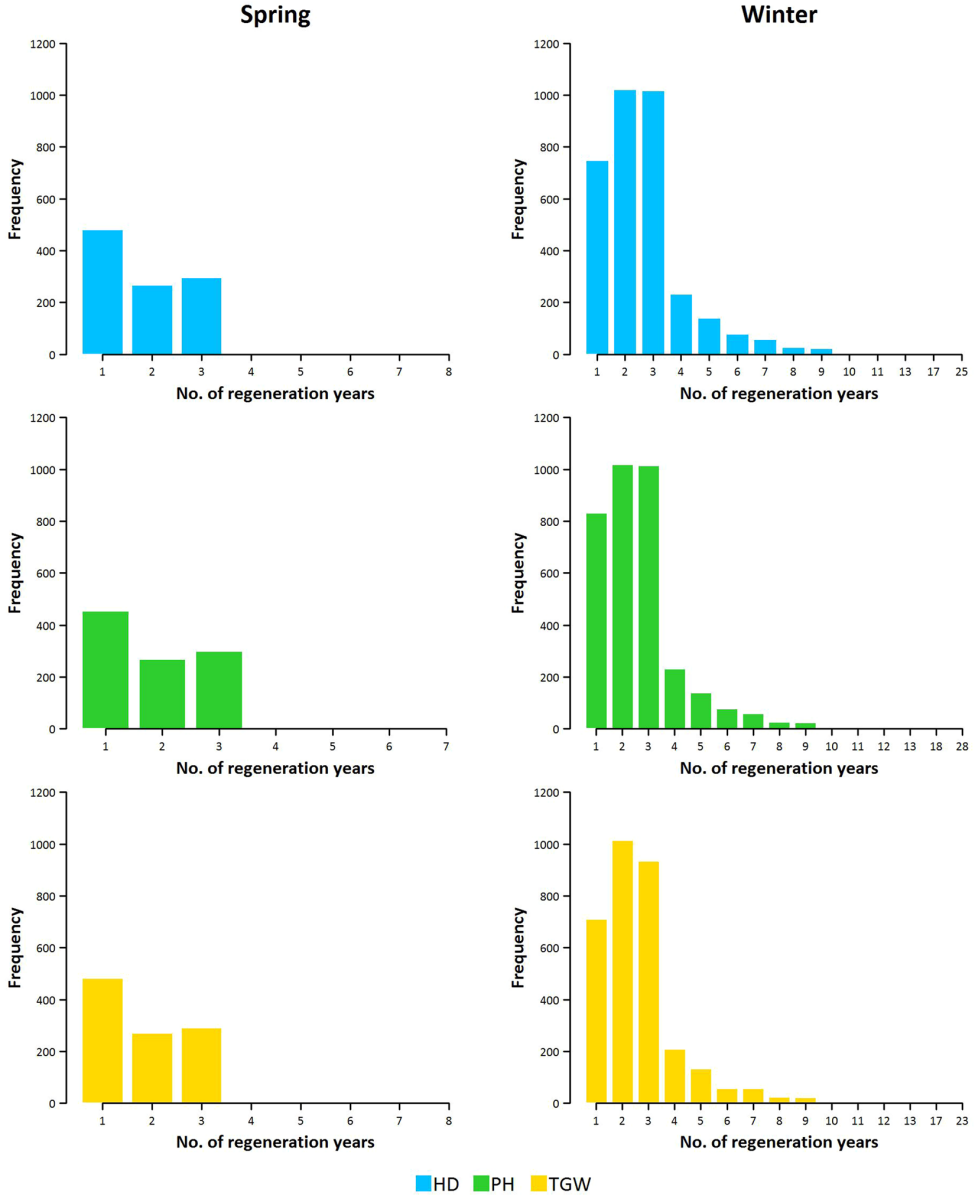
Number of regeneration years in which accessions of the spring and winter wheat collections maintained at the Crop Research Institute (CRI), Prague, Czech Republic, were tested for heading date (HD), plant height (PH), and thousand grain weight (TGW).

**Fig. 5:**
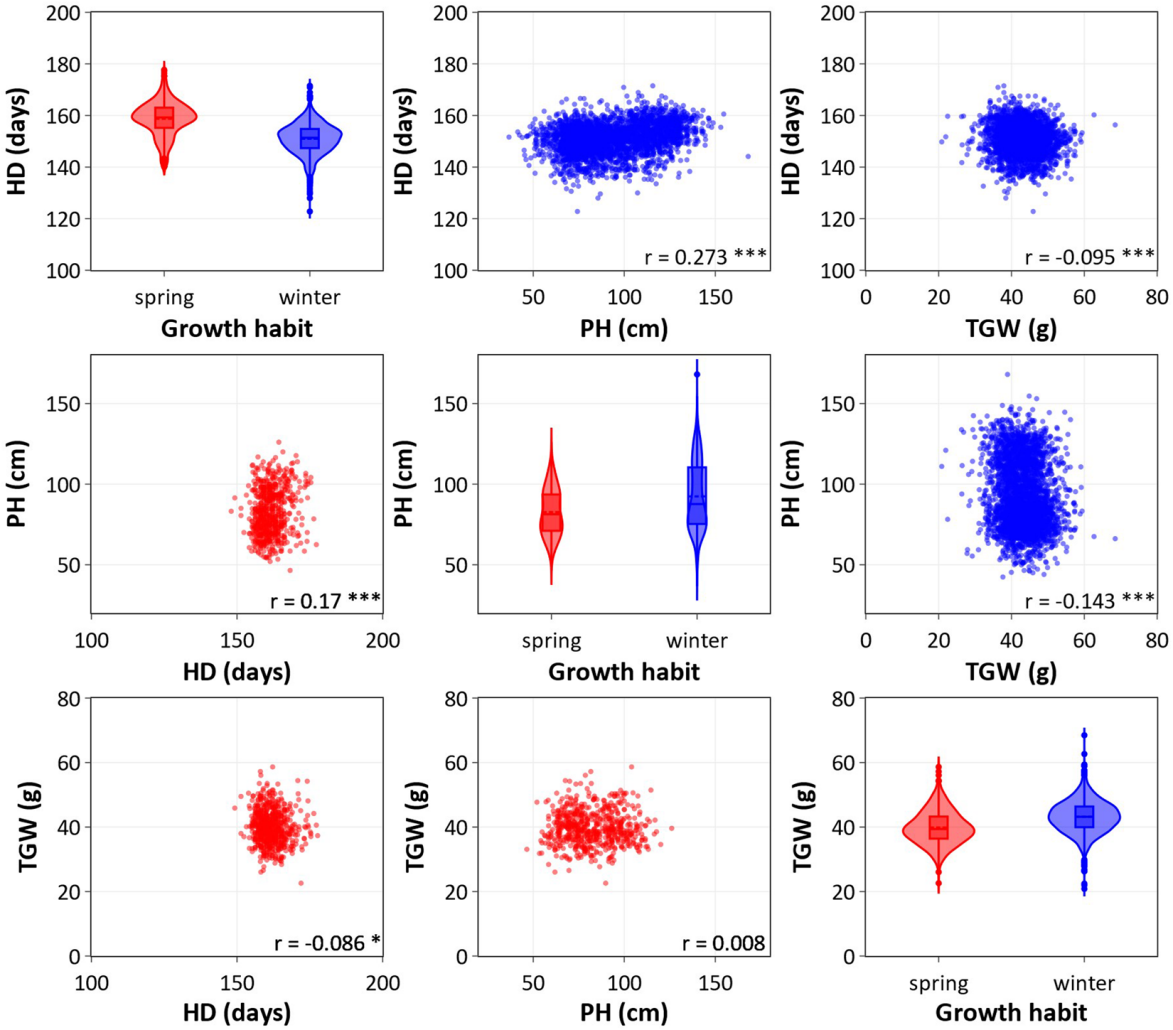
Matrix plot for the best linear unbiased estimations for HD (d), PH (cm) and TGW (g) as observed for 1,065 spring (in red) and 3,469 winter wheat accessions (in blue). Violin plots on the diagonal displays the dispersion of each trait of spring and winter wheat. Scatter plots in the upper and lower quadrant of the matrix shows the correlations among the three traits for spring and winter wheat, respectively. The Pearson’s correlation coefficients (r) which differ significantly from zero (P-value < 0.001) is indicated by ***.

The number of accessions tested for spring wheat increased up to 300 accessions in a single year (1975), as the numbers were comparable for all three traits (Fig. 3). Individual accessions were regenerated in 1 to 8 different years, with the most frequent values being 1 and 3 years (Fig. 4). For winter wheat, the number of accessions tested within individual years was even higher, with at least 500 accessions being tested in at least four different years. Individual accessions were regenerated in 25 (HD), 28 (PH), and 23 (TGW) different years, with the prevalence of a 2- and 3-year regeneration cycle. Additionally, the Best Linear Unbiased Estimations (BLUEs; Fig. 5) were provided for the accessions in the “BLUEs.spring.txt” and “BLUEs.winter.txt” files and were calculated using the enhanced historical data files.

**Fig. 6:**
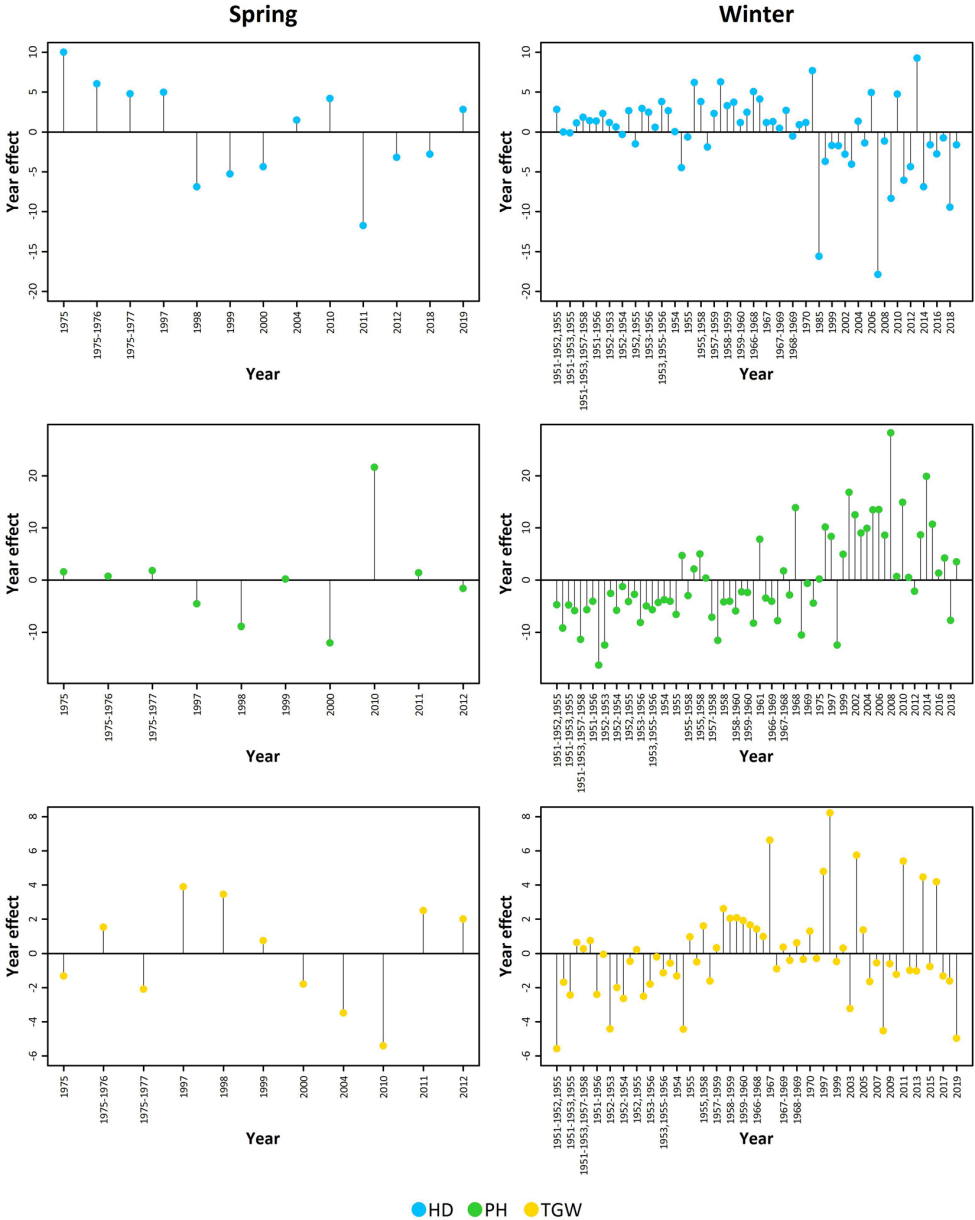
Effect of the year for heading date (HD), plant height (PH), and thousand grain weight (TGW) for spring and winter wheat. Enhanced dataset without outliers were used.

The strongest year effect was observed for PH followed by HD. TGW was the least affected. The same scenario applies to both spring and winter wheat (Fig. 6).

## Technical Validation

Validation performed in this study involves correction for the outliers in individual traits following the routines described by Phillip *et al*.^15^. Brief description of the methods follows.

### Outlier corrections to curate the data

Due to the diversity of our dataset, which spans seven different decades and includes data collected under varying climate conditions and seed regeneration cycles, the occurrence of outliers is to be expected. However, as these outliers can disrupt the statistical estimation of the data, it is essential to manage them properly. Dealing with outliers in such unbalanced historical datasets can be challenging. To address this issue, we employed an outlier inspection approach that combines the rescaled median absolute deviation of standardized residuals with a Bonferroni-Holm test to identify and flag data points as outliers. To do so, we established a predefined significance threshold of p-value < 0.05 for the implemented test and removed the identified outliers from the historical dataset, resulting in an enhanced dataset. After the removal of outliers, we re-fitted Eq. (1), treating genotypes and years as random effects, and evaluated the impact of outlier exclusion on variance components and heritability.

**Fig. 7:**
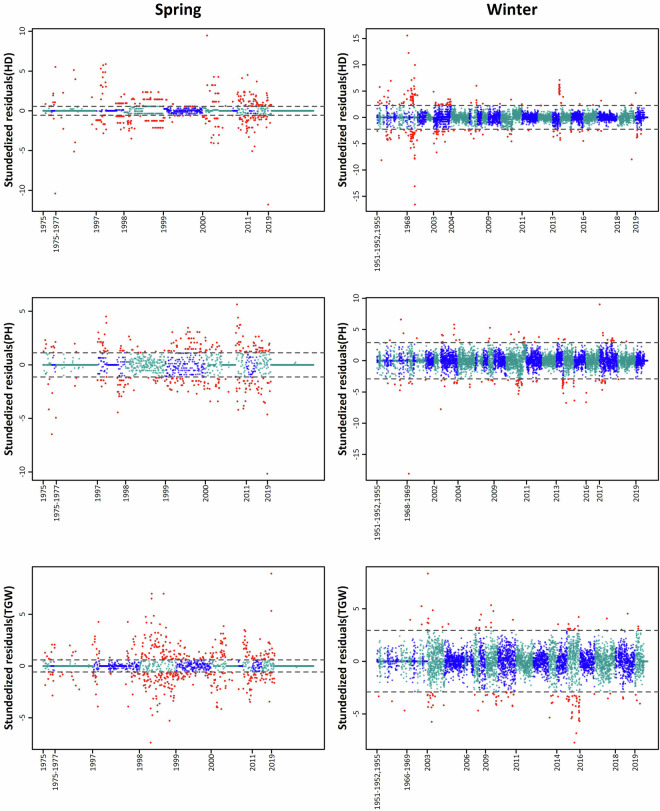
Studentized residuals for heading date (HD), plant height (PH), and thousand grain weight (TGW) of spring and winter wheat per regeneration years. Residuals were estimated by modeling independent variances of residuals for each regeneration year. Different colors are used to distinguish between different years. Dashed lines separate the outliers, which are highlighted in red.

The correction of outliers had a significant impact on the heritability (h²) of spring wheat, leading to an improvement of up to 24% compared to the original data (see Table 2). This improvement can be attributed to a reduction in error variance, which was particularly pronounced for spring wheat, where the original dataset contained a relatively high proportion of outliers (up to 26%; see Table 3 and Fig. 7). However, in the case of HD, the correction of outliers interfered with the model used, making it impossible to calculate the effect of outlier correction on h². As for winter wheat, outliers were present in only 3.5% of the data, resulting in a modest reduction in error variance and a smaller improvement in heritability (up to 10%; see Table 2).

**Table 2:** Variance components (σ²) and heritability for original and outlier corrected datasets for heading date (HD), plant height (PH), and thousand grain weight (TGW) for the spring and winter wheat accessions maintained at the Crop Research Institute (CRI), Prague, Czech Republic.

Growth habit	Data	Variable	HD	PH	TGW
Spring	Original	σ²_Genotypes_	16.73	192.28	23.2
		σ²_Years_	50.6	175.34	12.46
		σ²_Errors_	25.25	40.87	9.35
		σ²_Environment_	1.49	1.52	1.58
		h^2^	0.5	0.88	0.8
	Enhanced	σ²_Genotypes_	—	200.98	26.59
		σ²_Years_	—	97.39	10.62
		σ²_Errors_	—	5.54	0.32
		σ²_Environment_	—	1.62	1.59
		h^2^	—	0.98	0.99
Winter	Original	σ²_Genotypes_	23.27	297.27	14.96
		σ²_Years_	28.35	50.69	9.78
		σ²_Errors_	10.05	86.84	9.36
		σ²_Environment_	1.98	1.99	1.95
		h^2^	0.82	0.87	0.76
	Enhanced	σ²_Genotypes_	24.92	311.47	16.42
		σ²_Years_	27.28	49.79	9.46
		σ²_Errors_	5.46	87.38	7.52
		σ²_Environment_	1.93	1.97	1.93
		h^2^	0.9	0.88	0.81

**Table 3:** Number of records, accessions and outliers as recorded for heading date (HD), plant height (PH), and thousand grain weight (TGW) for the spring and winter wheat collections maintained at the Crop Research Institute (CRI), Prague, Czech Republic.

Growth habit	Trait	Number of records	Number of accessions	Number of records (after pruning)	Number of accessions (after pruning)	Number of outliers	Outliers (%) ratio to all data (after pruning)	corrected data size
Spring	HD	1,673	1,062	1,668	1,058	414	24.82	1,254
	PH	1,652	1,039	1,647	1,035	303	18.4	1,344
	TGW	1,810	1,061	1,805	1,057	466	25.82	1,339
Winter	HD	7,857	3,369	7,813	3,339	273	3.49	7,540
	PH	8,105	3,449	8,053	3,424	143	1.78	7,910
	TGW	7,115	3,184	7,071	3,154	126	1.78	6,945

## Usage Notes

The presented FAIR data for a further significant wheat collection are to be seen in the context of already published data^15–18^ and represent an important step towards a global catalog of plant genetic resource information, which is essential to transform genebanks into bio-digital resource centers^19^ and thus, support future research and breeding initiatives. Genebank material is available upon request and can be accessed through GRIN Czech platform under the terms of a Standard Material Transfer Agreement (SMTA).

## Data Availability

Described statistical approaches were performed within the R environment (version 4.0.3). Scripts used for the both outlier correction (“outlier.correction.R”) and estimation of BLUEs (BLUEs.estimation.R) along with input datasets containing original historical data can be found in the e!DAL-Plant Genomics and Phenomics Research Data Repository (PGP).To run the script, input data files (a_spring_wheat.txt; a_winter_wheat.txt) need to be downloaded and proper working directory needs to be set. The script named “outlier.correction.R” shows the outlier handling procedure on the example of plant height data in winter wheat. Output files such as “Var.comp.PH.txt”, “Outliers.PH.txt” and “Data.corrected.PH.txt” corresponding to variance components or regeneration years, list of removed outliers and enhanced (outlier-corrected) data, respectively are generated by the scripts. Script called “BLUEs.estimation.R” uses the outlier corrected data from previous step while generating the BLUEs for individual accessions, as included in the “BLUEs.PH.txt” output. As data for all traits are available in input files, script can easily be altered for other traits and spring wheat following the footnotes within script.
